# Cost-effectiveness analysis of locking nail compared with locking plate for displaced 3- and 4-part proximal humerus fractures: a secondary analysis of a randomized trial comparing the Multiloc nail and PHILOS plate

**DOI:** 10.2340/17453674.2025.44881

**Published:** 2025-10-27

**Authors:** Annette Konstanse Bordewich WIKERØY, Per-Henrik RANDSBORG, Eline AAS, Hendrik Frølich Stange FUGLESANG, Rune Bruhn JAKOBSEN

**Affiliations:** 1 Orthopaedic Department, Akershus University Hospital, Akershus; 2 University of Oslo, Institute of Clinical Medicine, Oslo; 3 University of Oslo, Department of Health Management and Health Economics, Oslo, Norway

## Abstract

**Background and purpose:**

Previous studies show no clear difference in functional outcomes between locking nails and plates for proximal humerus fractures (PHFs). Economic evaluations provide valuable insights into cost-effectiveness to guide healthcare decisions. We aimed to conduct a cost-effectiveness analysis based on a semidouble-blinded randomized controlled trial comparing nailing and plating for displaced 3- and 4-part PHFs with 2-year follow-up.

**Methods:**

79 patients with displaced 3- or 4-part PHFs were randomized to undergo open reduction and internal fixation using either a nail or a plate. Patients were followed for 2 years, with costs tracked for the index surgery, hospital stay, additional healthcare services, and secondary procedures. Quality-adjusted life years (QALYs) were calculated using EQ-5D, and incremental cost-effectiveness ratios (ICERs) were used to compare treatments.

**Results:**

38 patients in each group were eligible for analysis. Mean total costs were €21,654 (standard deviation [SD] 10,448) for nails and €16,374 (SD 3,886) for plates, with a mean difference of €5,296 (95% confidence interval [CI] 1,989–8,603). Extra costs for reoperations and other non-regular follow-ups were €3,746 (SD 10,448) for nails and €265, (SD 1,217) for plates, resulting in a mean difference of €3,480 (CI –868 to 7,829) extra costs for nails. The mean QALY showed no statistical difference between groups of 0.09 (CI –0.003 to 0.17) (1.65 in the nail group and 1.74 in the plate group).

**Conclusion:**

Plates were more cost-effective compared with nails but did not result in a statistically significant difference in QALY.

Proximal humerus fractures (PHFs) rank as the third most common fractures among the elderly, their incidence tripling over the past 3 decades [1]. These fractures pose a significant burden to the elderly, causing pain, functional impairment, and even increased mortality. They have societal implications, including higher healthcare costs and, in some cases, loss of independent living [2,3].

Recent high-quality studies comparing nonoperative and surgical treatments have shown that surgical intervention offers no clear advantages in terms of patient function, quality of life, or cost-effectiveness for the elderly [4-6]. Despite this, surgical intervention is still considered for some fractures due to factors such as fracture morphology, the patient’s overall health, age, and compliance ability. Economic evaluation is a crucial tool for guiding healthcare decisions, providing insights into both effectiveness and cost-effectiveness. We previously conducted a semidouble-blind randomized controlled trial (RCT) comparing locking nails with locking plates for displaced 3- and 4-part PHFs [7]. The study revealed no difference in functional outcomes between the implants, aligning with previous literature [8]. However, after 2 years, nailing was associated with higher rates of complications and reoperations. Given the comparable functional outcomes, cost considerations may play a decisive role in treatment selection. There is limited research on the cost-effectiveness of PHF treatments. The Cochrane Collaboration has highlighted the importance of integrating resource assessments into this field, enabling decision-makers to evaluate the value for money of various interventions [6].

The aim of this study is to evaluate the cost-effectiveness based on the previously conducted RCT of surgical treatment with the Multiloc nail compared with the PHILOS plate (both manufactured by Synthes, Raynham, MA, USA). The primary outcome was quality-adjusted life years (QALYs), and secondary costs assessed from a healthcare perspective over a 2-year period. We hypothesized that plates would demonstrate superior cost-effectiveness compared with nails.

## Methods

This study is reported according to the CONSORT guidelines.

### Study design

The patients were recruited from and treated at Akershus University Hospital in Norway. Table 1 shows the inclusion and exclusion criteria. Between October 2016 and June 2021, 130 patients aged between 18 and 85 presented to our institution with a displaced 3- or 4-part PHF. 79 patients fulfilled inclusion criteria and were randomized using a computer-generated permuted block randomization to either Multiloc nail or PHILOS plate. The age range of patients included was 43 to 81 years, mean 67 years. Though registered as a 5-year trial to allow long-term reporting, the primary endpoint was the 2-year DASH score. Enrollment was halted at 79 patients—below the initial target of 90—due to minimal loss to follow-up. This probably reflects the single-center design, close monitoring by the first author, and a stable, compliant population with high trust in the healthcare system. The missing data and sensitivity analysis is published as an appendix to the RCT [7].

**Table 1:** Inclusion and exclusion criteria

Inclusion criteria
● Patients between 18 and 85 years
● Displaced a 3- or 4- part PHF
● Type 11B1.1, 11B1.2, 11C1.1, 11C1.1p (OTA/ AO 2018 revision)
Exclusion criteria
● Illness or injury that will influence recovery and scoring systems
● Open fractures
● Fracture dislocation
● Head split fractures
● Neurovascular injury or disease
● Multitrauma/ high energy or “multifractured patient”
● Substance or alcohol abuse
● Dementia or non-compliance
● Inability to read and understand Norwegian
● Patients not residing in our catchment area
● Ongoing infectious disease
● Pathological fracture
● Fracture more than 3 weeks old
● Humeral shaft diameter too narrow for nailing
● Any medical condition that excludes surgical treatment, including patients with ASA 3 or ASA 4 who are considered too ill to go through surgery

a Displacement defined as ≥ 45° valgus or ≥ 30° varus in a true anteroposterior projection, 45° angulation in a scapular Y projection, or > 50% displacement of the humeral head against the shaft.

OTA = Orthopaedic Trauma Association; AO = Arbeitsgemeinschaft fur Osteosynthesefragen; PHF = fracture of the proximal humerus; ASA = Physical Status Classification System, p = both tubercles fractured according to OTA/AO classification of humerus fractures.

### Surgery

Surgery was performed within 3 weeks after injury by 2 experienced trauma surgeons trained in both implants and instruments. This was carried out under general anesthesia with additional regional anesthesia and antibiotic prophylaxis. All patients had an anterolateral/deltoid split, except 2 with deltopectoral. The anterolateral approach is the approach most used in our institution. The tuberosities were fixed with non-absorbable sutures tied to the PHILOS plate or the Multiloc “gold” screws. Patients were offered a sling for comfort for a maximum of 2 weeks. All patients started self-exercises on the first postoperative day. Physiotherapists instructed the patients in a self-exercise program with exercises without resistance for the first 6 weeks and recommended outpatient physiotherapy starting 2 weeks after discharge. There was no study-specific physiotherapy program. The patients were followed up for 6 weeks, 3 and 6 months, and 1 and 2 years. Return to work was a joint decision between the patient, surgeon, and the general practitioner.

### Outcomes

The primary clinical outcome was the Disabilities of Arm, Shoulder and Hand (DASH) score 2 years after surgery. The DASH score is a PROM questionnaire with 30 questions. It has been translated into Norwegian and validated. The scores range from 0 (perfect extremity) to 100 (completely disabled limb) [9,10]. The minimally clinically important difference was estimated to be 10 points [11-14], and the standard deviation (SD) was assumed to be 15 [15]. With a significance level of 0.05 and an 80% power, the sample size calculation indicated a need for 36 patients in each group. Initially we postulated a 20% loss to follow-up, needing to include 90 patients, but inclusion was terminated at 79 patients because of minimal loss to follow-up. A full description of the intervention and clinical results has been reported previously [7].

#### Health related quality of life/effect measurement

The EQ-5D-3L is a validated generic instrument for the measurement of health-related quality of life (HRQoL) that allows the translation of patient HRQoL scores to quality-adjusted life years (QALYs) [16,17]. It is the instrument recommended by the Norwegian Institute of Public Health [18] and the National Institute for Health and Care Excellence (NICE) technology appraisal guidance [19]. According to Stavem et al., the mean normative HRQoL in Norwegians aged between 61 and 70 years is 0.82, SD 0.21 [20]. Evaluation of EQ-5D-3L with regards to PHFs has shown that EQ-5D-3L displayed good internal and external responsiveness and can be recommended as an HRQoL measure in clinical studies and healthcare assessments [21]. QALY is a generic measure of disease burden and combines HRQoL and time, a measure anchored at 0 (death) and 1 (perfect health) [22]. 1 year in perfect health equals 1 QALY. The EQ-5D-3L measures health status in 5 dimensions: mobility, self-care, usual activities, pain/discomfort, and anxiety/depression, each with 3 response levels: no problems, moderate problems, and severe problems. EQ-5D-3L was obtained at baseline, 6 weeks, 3 and 6 months, and 1 and 2 years. We then estimated HRQoL using a valuation algorithm derived from a general population study in Denmark [23]. QALYs were estimated using the trapezoidal method (area under the curve) [24], and the incremental effect was the difference in QALYs between the 2 groups.

#### Costs

A cost-per-patient system (CPP) was used to register costs prospectively during hospital stay including personnel use, time spent in the operation theatre, postoperative care unit, orthopedic ward, and overhead costs. These cost components were used for in-hospital resources both for the index stay, readmissions, outpatient visits, and telephone consultations. All costs are presented in 2023 prices converted to euro, using the exchange rate €1 = NOK8.6.

#### Primary healthcare utilization

At each time point (6 weeks, 12 weeks, 1 year, and 2 years), patients were asked by their doctor or physiotherapist to report, via questionnaires, how many times they had visited a physiotherapist, consulted a general practitioner, or received home nursing and home care services since their last outpatient clinic visit. Valuation was collected from the Norwegian Health Economics Administration (HELFO). Sick leave was registered but not included in the economic calculations.

Table 2 present the included healthcare utilization, units, valuation, and sources. Costs were divided into 3 categories: (i) hospital costs for the index admission/surgery, (i) additional healthcare service-related costs outside the hospital, and (iii) costs related to additional surgical procedures.

**Table 2:** Resources used and their cost during treatment of proximal humerus fracture, numbers in euro, 2023

Cost category	Unit	Unit cost	Source
Hospital			
Equipment			
Plate including standard number of screws **^a^**	Operations	958	Real costs
Nail including standard number of screws ^b^	Operations	619	Real costs
Inpatient costs, surgery cost inpatient			CPP
2 orthopedic surgeons (knife time)	Minutes	13.9	
Anesthesiology (incl. nurse, anesthesiologist)	Minutes	8.9	
Operation theatre (incl. nurses and consumables)	Minutes	12.9	
Postoperative ward	Minutes	3.0	
Orthopedic ward	Days	855	
Cost reoperation day surgery			CPP
2 orthopedic surgeons (knife time)	Minutes	14.3	
Anesthesiology (incl. nurse, anesthesiologist)	Minutes	11.3	
Operation theatre (incl. nurses and consumables)	Minutes	6.1	
Postoperative ward	CPE	518.2	
Radiology			BDA
Radiographs	Per exam	129	Real costs
Magnetic resonance imaging	Per exam	439	Real costs
Computer tomography	Per exam	215	Real costs
Distension arthrography	Per exam	387	Real costs
Costs related to outpatient care			CPP
Outpatient clinic consultation	20 min	213.3	
Telephone consultation	20 min	213.3	
Costs after discharge, rehabilitation			
Physiotherapy first visit	CPE	66.6	NPU
Physiotherapy supplementary visit	CPE	38.1	NPU
Consultation GP index visit (removal stitches)	CPE	95.8	DMP
Consultation GP (cont. sick leave)	CPE	58.0	DMP
Home nursing	30 min	25.2	c
Home care	60 min	27.9	c

All prices are in euro; Norwegian Kroner multiplied by an exchange rate of 0.086.

a Philos plate with 1 cortical screw and 11 locking screws.

b Multiloc nail with 3 Multiloc/gold screws and 2 locking screws. In the analysis prices are adjusted according to actual number of screws used.

c Ministry of Local Goverment and modernization/ Hammer et al. [38]

BDA = radiology department at Ahus, CPE = cost per episode, CPP = cost per patient; including information oncosts during surgery, in the different hospital wards, implant costs, administration, and other overhead costs. DMP = Norwegian medical products agency, GP = general practitioner, NPU = Norwegian Physiotherapy Union.

#### Cost-utility analyses

We report the result from the cost-utility analysis as the incremental cost-effectiveness ratio (ICER), a measure that reflects the incremental costs (ΔCosts) relative to incremental QALYs (Δ QALYs), see Equation (1). The ICER can be interpreted as the extra costs per 1 QALY.


$$\mathrm{ICER}=\frac{{\mathrm{Costs}}_{\mathrm{Nail}}-{\mathrm{Costs}}_{\mathrm{Plate}}}{{\mathrm{QALYs}}_{\mathrm{Nail}}-{\mathrm{QALYs}}_{\mathrm{Plate}}}=\frac{\mathrm{\Delta Costs}}{\mathrm{\Delta QALYs}}$$ (1)

The ICER is evaluated in reference to a cost-effectiveness threshold (CET), defined as the maximum value a payer is willing to spend per additional QALY. A treatment is considered cost-effective if the ICER is below the CET. In Norway, the CET depends on the severity of the condition (measured by absolute shortfall) and varies from NOK 275,000 = €31,977 to NOK825,000 = €95,930 (2023) [25].

### Statistics

There were no missing values in the cost estimates. Missing values in the HRQoL data at baseline were imputed using the mean value for all patients. For subsequent time points, multiple imputation by chained equations (MICE) was used. Cumulative values were then estimated from the imputed datasets.

Because cost data were non-Gaussian, we tested for significant differences in costs by means of regression analysis with use of a generalized linear model with a gamma distribution and a log-link function [22], adjusted and unadjusted for baseline HRQoL, and calculated the 95% confidence intervals (CI) for the mean difference.

We assessed the uncertainty in the ICER by bootstrapping costs and effects with 1,000 iterations. Results are presented with use of cost-effectiveness planes. Statistical analyses were performed using STATA 18.0 (StataCorp LLC, College Station, TX, USA), Microsoft Excel 16 (Microsoft Corp, Redmond, WA, USA, and Graph Pad Prism 10 (https://www.graphpad.com/features).

### Ethics, data sharing plan, funding, use of AI, and disclosures

The study was conducted according to the Declaration of Helsinki and approved by the Regional Ethics Committee of Southeastern Norway (2016/ 626) and the local data protection officer (PVO 2016_061). The RCT (ref) was registered at www.Clinicaltrials.gov (NCT02944058). Written informed consent was obtained from all patients.

The data is available on reasonable request.

Copilot has occasionally been used for translation from Norwegian to English, not for text generation, art, programming, statistical analysis, or other AI assistance. The trial was funded by Akershus University Hospital, Sofies Minde AS, and the Norwegian Orthopaedic Association. Disclosure of potential conflicts of interest forms are provided with the online version of the RCT (http://links.lww.com/JBJSOA/A754) and on the article page, doi: 10.2340/17453674.2025.44881

## Results

**Figure 1:**
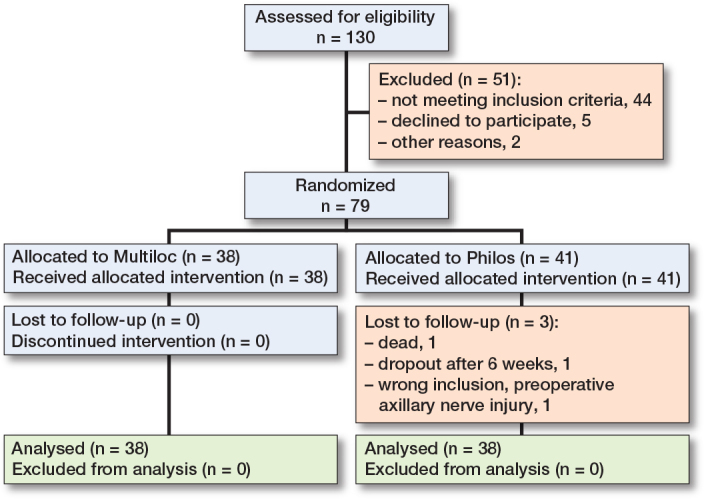
Flowchart of patient inclusion

130 patients were assessed for eligibility in the trial, 44 did not meet the criteria for inclusion, 5 declined to participate, 2 were discovered to meet inclusion criteria during surgery (they were already excluded for some reason and not randomized, see CONSORT), leading to 79 patients being randomized. At the 2-year follow-up, there were 38 patients in each group. The mean age of included patients was 67 years (range 43–81) and 66 (84%) patients were women. Table 3 presents baseline characteristics in the 2 groups. 3 were lost to follow-up; 1 died (cause unrelated to the study), 1 dropped out, and 1 was later discovered to be wrongly included (Figure 1).

**Table 3:** Baseline characteristics of 79 patients with a proximal 3- or 4-part fracture of the proximal humerus, allocated to either intramedullary nail or plate fixation

Characteristic	Nail (n = 38)	Plate (n = 38)
Women, n	33	31
Age at surgery, mean (SD)	66 (9)	67 (10)
Smokers, n	3	5
Diabetes mellitus, n	2	2
Body mass index, mean (SD)	27 (4)	29 (5)
Days from trauma to surgery, mean (SD)	6 (3)	8 (5)
Fracture of dominant side, n	20	14
Duration surgery, minutes, mean (SD)	101 (23)	93 (22)
HSA of healthy shoulder, mean (SD)	141° (5°)	141° (6°)
range	127°–155°	131°–160°
Baseline DASH, mean (SD)	1.5 (3.6)	1.0 (2.3)
Neer classification, n		
3-part	23	27
4-part	15	11
Displacement, mm, mean (SD)		
greater tuberosity	20 (9)	18 (8)
range	8–45	8–37
lesser tuberosity	14 (5)	12 (4)
range	9–23	10–19
AO/OTA classification, n		
11B1.1/ 11B1.2	20	23
11C1.1	3	4
11C1.1p	15	11

AO = Arbeitsgemeinschaft fur Osteosynthesefragen;

DASH = Disability of the Arm, Shoulder and Hand score;

HSA = head shaft angle; OTA = Orthopaedic Trauma Association; SD = standard deviation.

14 (37%) patients in the nail group and 4 (11%) patients in the plate group experienced 1 or more major complications. 12 (32%) patients in the nail group and 2 (5%) patients in the plate group were reoperated on. 1 patient in the nail group was found to have poor-quality fracture reduction but declined reoperation. Patients who developed avascular necrosis (AVN) reported markedly worse outcomes than patients without AVN. 6 patients in the nail group developed AVN. All 6 patients had their implants removed. 1 patient suffered a humeral shaft fracture during the follow-up period and underwent re-nailing, and 1 patient had an infection and was revised. 2 patients in the plate group were offered implant removal or arthrolysis because of AVN but declined surgery. These complications were considered major, despite not leading to reoperation. A third patient underwent arthrolysis and plate removal. The patient was later scheduled for arthroplasty, but the surgery was cancelled because of an unrelated acute illness. A sensitivity analysis was performed excluding the patient with complications, which did not affect the primary outcome.

### Health-related quality of life

At the time of inclusion, the mean HRQoL was 0.94 in both groups as measured by EQ5D.

**Figure 2:**
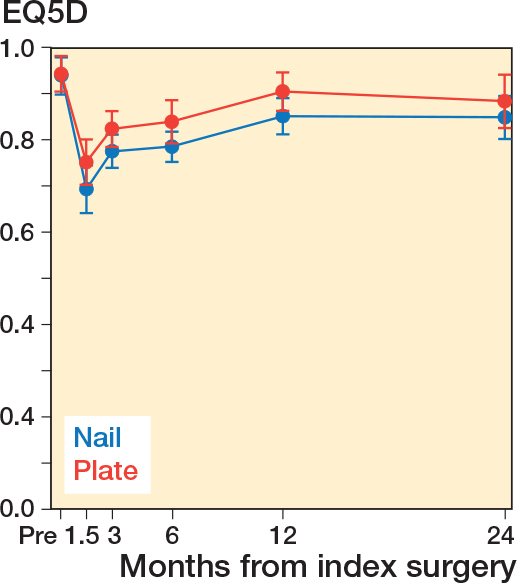
HRQoL measurements comparing nails (blue lines) with plates (red lines) from pre-injury to 2 years’ follow-up.

Generally, the HRQoL measurements were lower in the nail group than in the plate group at all time-points, but at no time statistically significantly different (Figure 2). After 2 years’ follow-up, the mean QALYs were 1.65 (CI 1.44–1.86) for the nailing group and 1.74 (CI 1.50–1.99) for the plating group, resulting in a statistically non-significant difference between groups of 0.09 (CI –0.003 to 0.17).

### Resource utilization and costs

There were no statistically significant differences between the 2 groups regarding surgical time or length of stay for the index hospital stay, but there were more reoperations and complications in the nail group (Table 4). Table 5 shows mean costs per patient stratified between the 2 treatment groups.

**Table 4:** Complications and reoperations stratified by implant

Factor	Nail (n = 38)	Plate (n = 38)	P value
Patients with complications, n (%)	14 (37)	4 (11)	0.05
Total number of complications	20	10	
AVN	6	3	
Infection	3	0	
Insufficient reduction	1	0	
Absorption of major tubercle	3	2	
Loss of reduction tubercle	1	1	
Pain without known cause	1	1	
Frozen shoulder	2	1	
Cut-out of screw/screw loosening/protruding screw	2	2	
Re-fracture after metal removal	1	0	
Patients with reoperations, n (%)	12 (32)	2 (5)	0.006
Total number of reoperations	15	2	
Removal of metal			
AVN	4	1	
Cut-out of screw/screw loosening/ screw too long	2	2	
Infection (arthroscopic debridement and removal of metal)	2	0	
Pain without obvious reason	0	1	
Arthrolysis/revision /debridement			
Infection	1	0	
Frozen shoulder	2	0	
Stiff shoulder	3	0	
Re-fracture after metal removal, re-nailing	1	0	

Patients could have more than 1 complication or reoperation.

P values calculated with Fisher’s exact test.

HSA = head shaft angle; AVN = avascular necrosis of humeral head.

**Table 5:** Mean costs in euro stratified by treatment groups

	Nail (n = 38)	Plate (n = 38)	Mean difference (CI)
Initial hospital cost			
Ward	4,997	4,119	877 (–191 to 1,947)
Surgery			
Time in operation theatre	2,779	2,633	146 (–17 to 309)
Knife time	1,373	1,303	70 (–64 to 204)
Time anesthesia	1,73	1,601	129 (19 to 240)
Time postoperative ward	560	512	47 (–94 to 190)
Initial radiology	473	473	0 n/a
Cost implant **^a^**	1,21	1,401	–191 (–265 to –117)
Total initial hospital cost	13,123	12,042	1,081 (–109 to 2,271
Costs related to outpatient care			
Consultations outpatient clinic (incl. phone consultations)	1,154	974	179 (–1 to 360)
Home nursing, Homecare, GP, outpatient physiotherapy	2,589	2,134	455 (–440 to 1,350)
Radiology (radiographs, CTs, MRIs)	1,043	960	83 (–50 to 216)
Total outpatient care cost	4,785	4,068	717 (–252 to 1,687)
Reoperations and other non-regular follow-ups **^b^**			
Ward	1,954	113	1,841 (–677 to 4,361)
Surgery (combined OR time/ surgeon/ anesthesia)	1,748	153	1,595 (–311 to 3,502)
Cost implant **^b^**	44	0	44 n/a
Total reoperation cost	3,746	265	3,480 (–868 to 7,829)
Total cost, unadjusted	21,653	16,374	5,279 (1,997 to 8,561)
Total cost, adjusted	21,663	16,367	5,296 (1,989 to 8,603)

a Includes costs of one-time-use drill bits, cement if used, additional locking screws, endcaps, and other one-time use implant equipment

b 2 patients in the nail group changed implant, the total price of the 2 implants was €1,627; there were no reoperation implant costs in the plate group.

CI = 95% confidence interval, CT = computed tomography; GP = general practitioner; MRI = magnetic resonance imaging; OR: operation room; n/a: not applicable.

The mean costs for the index hospital stay were €13,123 (SD 3,093) for the nail group and €12,042 (SD 2,180) for the plate group, with a mean difference of €1,081 (CI –109 to 2,271). While surgical time for nail procedures was longer, the difference was not statistically significant. However, when cost components such as OR time, knife-to-skin time, and anesthesia duration are priced, the cumulative theatre costs for nail procedures become higher. Similarly, the mean hospital stay was slightly longer for the nail group (5.8 days, SD 3.4) than for the plate group (4.8 days, SD 2.2), though this too was not statistically significant. These small, non-significant differences likely explain the observed cost variation. Similarly, total outpatient-related costs were €4,785 (SD 1,877) for the nail group and €4,068 (SD 2,293) for the plate group, with a mean difference of €717 (CI –252 to 1,687). However, patients in the nail group incurred significantly higher costs associated with reoperations. The mean total reoperation cost per patient was €3,746 (SD 8,749) for the nail group compared with €265 (SD 1,217) for the plate group resulting in a mean difference between treatments for the total costs of €5,279 (CI 1,997–8,561) Hence, the differences in costs for the 2 treatments are due to complications and reoperations. For the additional costs of reoperations for each patient, see Appendix. The average number of sick-leave days was 32 days (SD 62) for the nailing group and 19 days (SD 45) for the plating group.

### Cost-effectiveness analysis

**Figure 3:**
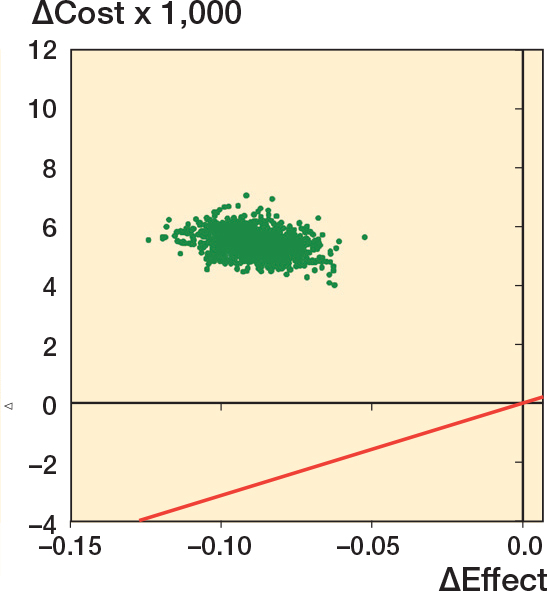
Cost-effectiveness plane demonstrating the bootstrapped cost differences of the ICER between nailing and plating from a healthcare perspective with a cost-effectiveness threshold (CET) per QALY (red line). The scatterplot illustrates that plating is both more effective and less costly than nailing. CET= NOK 275,000 = €31,977.

Figure 3 presents the cost-effectiveness plane on bootstrapped ICER values, demonstrating that plates are more effective and less costly than nails (plates dominate nails). As all bootstrapped values show that nailing is consistently more expensive and less effective, cost-effectiveness acceptability would be absolute in favor of plating regardless of any chosen CET.

A sensitivity analysis removing all patients with reoperation from the full analysis reduced the cost difference between the groups from €5,279 to €1,623, still in favor of the plating group, albeit with CI crossing €0 (–100 to 3,347).

## Discussion

This is the first study directly comparing the QALY and cost-effectiveness of locking plates vs locking nails in complex PHFs. We aimed to evaluate the cost-effectiveness based on the previously conducted RCT of surgical treatment with the Multiloc nail compared with the PHILOS plate for patients aged 18 to 85 years with displaced 3- or 4-part PHFs. After 2 years of follow-up, there was no statistically significant difference in QALYs between the plate and nail groups. However, locking plates were more cost-effective than locking nails for the treatment of proximal humerus fractures. Although HRQoL scores were consistently lower in the nail group across all time points, these differences did not reach statistical significance. Our previously published RCT on the same patients showed equivalent functional outcomes for these 2 implants over a 2-year period, as assessed by the DASH score [7]. Our findings indicate that the cost difference is primarily driven by reoperations. However, even when reoperation costs are excluded, plates trend towards being more cost-effective than nails. This suggests minimal justification for choosing nails over plates at any stage of treatment.

3 randomized trials and 1 non-randomized trial have compared the functional outcomes and complication rates between these 2 treatment options [26-29]. Our findings are consistent with the RCT conducted by Gracitelli et al. [27], comparing nails with plates in 2- and 3-part PHFs. That study also reported a higher rate of complications and reoperations in the nail group; however, second-generation nails with a lateral entry point were used. The Multiloc nail is a third-generation nail with supposedly improved design aimed at enhancing fixation stability, though we did find significantly higher complication rate. In contrast, Plath et al. found significantly less failure and screw cut-out in nails in their level 1 RCT comparing nails with plates in 2-, 3-, and 4-part PHFs [26]. Reasons for this difference might be due to several limitations, including a follow-up period of only 1 year, a small sample size (55 patients), and the inclusion of fractures with a wide range of complexity. Konrad et al. showed similar outcomes, complications, and reoperations for nail and plate fixation of 3-part PHFs [28]. They concluded that complications were related to incorrect surgical techniques rather than type of implant; however, the non-randomized design of that study likely biased that conclusion. In our study 2 experienced trauma surgeons performed all surgeries, so one could argue that incorrect surgical technique is less likely.

Boyer et al. reported higher complications and revision rates with locking plates compared with intramedullary nails [29]. However, the locking plates (Surfix; Surfix SAS, Lyon, France) used in their study featured fewer locking screw holes for the humeral head and lacked a calcar screw option. Several studies have emphasized calcar screws to be critical for enhancing the stability and outcomes of surgical treatments for proximal humerus fractures [30-32].

Non-surgical treatment of displaced PHFs is increasingly adopted in orthopedic trauma care due to a growing body of literature supporting its viability [4,5,33-35]. Although studies exploring the health economic implications of this trend remain scarce, the few available suggest no obvious cost effectiveness of surgery compared with nonoperative treatment of PHFs [36,37]. There are, however, essential differences in design and population in these 2 trials, limiting direct comparison with costs in our study.

### Strengths

A key strength of the current study is its comprehensive bottom-up approach to cost estimation. This includes detailed analysis of costs associated with the index surgery and hospital stay, additional healthcare services outside the hospital, and secondary surgical procedures. A cost per patient system enabled us to track the cost of each patient separately. The transparent breakdown of resource utilization and unit costs allows for clear identification of cost drivers, adding robustness to the findings. We chose not to include a societal perspective in the economic analysis, as sick-leave costs vary with system and country. Sick-leave in our population of retired or close-to-retirement patients was numerically higher in the nail group, and thus an analysis including sick-leave would have further substantiated dominance of plating over nailing.

### Limitations

Results from a single hospital may not have high external validity. However, the hospital has a large catchment area that includes both rural and urban areas, suggesting that the results are generalizable to other similar hospitals. The findings may not be representative of broader healthcare systems, making it difficult to generalize the economic outcomes to other regions or countries. When evaluating the surgical cost-effectiveness of different implants, it is essential to account for variations in costs across healthcare settings and trials. Factors such as local agreements, market dynamics, and resource utilization can greatly influence these costs, underscoring the importance of context-specific economic analyses. These variations can significantly impact external validity, highlighting the need to interpret findings within the study’s context and consider their applicability to other healthcare systems with caution.

### Conclusion

We found no statistically significant difference in QALY after 2 years between the groups. However, locking plates were significantly more cost-effective compared with nails.

*In perspective*, from a health economic perspective, plates dominate nails—being both more effective and less costly, as the term “dominate” implies. These findings make the routine use of nails in 3- and 4-part PHFs in clinical practice difficult to justify, particularly in settings with similar cost structures to our study.

## Notes

Conceptualization and study design: PHR, RBJ, AKBW, HSFF. Inclusion, surgery and data collection: AKBW, HSFF. Statistical and health economic analysis: EA, RBJ. First draft: AKBW. Revision: all authors.

Handling co-editors: Jeppe Vejlgaard Rasmussen and Robin Christensen

*Acta* thanks Carl Ekholm and Ville Mattila for help with peer review of this manuscript.

## Appendix

**Appendix 1:** Additional costs of reoperations

Patient number	Group	Additional total cost of reoperations (€)
4	Plate	3,202
12	Nail	9,104
15	Nail	30,416
28	Nail	4,797
31	Nail	18,473
35	Nail	3,925
46	Nail	27,328
47	Nail	34,097
66	Nail	3,992
68	Nail	5,495
74	Nail	4,707
79	Plate	6,869

## References

[1] Launonen A P, Lepola V, Saranko A, Flinkkilä T, Laitinen M, Mattila V M. Epidemiology of proximal humerus fractures. Arch Osteoporos 2015; 10: 209. doi: 10.1007/s11657-015-0209-4. 25675881

[2] Sumrein B O, Berg H E, Launonen A P, Landell P, Laitinen M K, Felländer-Tsai L, et al. Mortality following proximal humerus fracture: a nationwide register study of 147,692 fracture patients in Sweden. Osteoporos Int 2023; 34: 349-56. doi: 10.1007/s00198-022-06612-7. 36435907 PMC9852167

[3] van Eck C F, Klein C M, Rahmi H, Scheidt K B, Schultzel M, Lee B K, et al. Morbidity, mortality and cost of osteoporotic fractures: should proximal humerus fractures be taken as seriously as hip fractures? Ann Joint 2019; 4. doi: 10.21037/aoj.2019.01.01.

[4] Launonen A P, Sumrein B O, Reito A, Lepola V, Paloneva J, Jonsson K B, et al. Operative versus non-operative treatment for 2-part proximal humerus fracture: a multicenter randomized controlled trial. PLoS Med 2019; 16: e1002855. doi: 10.1371/journal.pmed.1002855. 31318863 PMC6638737

[5] Rangan A, Handoll H, Brealey S, Jefferson L, Keding A, Martin B C, et al. Surgical vs nonsurgical treatment of adults with displaced fractures of the proximal humerus: the PROFHER randomized clinical trial. JAMA 2015; 313: 1037-47. doi: 10.1001/jama.2015.1629. 25756440

[6] Handoll H H, Elliott J, Thillemann T M, Aluko P, Brorson S. Interventions for treating proximal humeral fractures in adults. Cochrane Database Syst Rev 2022; 6(6): CD000434. doi: 10.1002/14651858.CD000434.pub5. 35727196 PMC9211385

[7] Wikerøy A K B, Fuglesang H F S, Jakobsen R B, Thomas O M T, Randsborg P H. Intramedullary nail versus locking plate for displaced 3- and 4-part fractures of the proximal humerus: two-year results from a semidouble-blind randomized trial. JB JS Open Access 2025; 10(1): e24.00078. doi:10.2106/jbjs.Oa.24.0078. PMC11884838 40062004

[8] D’Almeida S S, Cannon R, Vu N T, Ponce B A, Redden D. Comparing intramedullary nails and locking plates in displaced proximal humerus fracture management: a systematic review and meta-analysis. Cureus 2024; 16: e54235. doi: 10.7759/cureus.54235. 38496197 PMC10944142

[9] Hudak P L, Amadio P C, Bombardier C. Development of an upper extremity outcome measure: the DASH (disabilities of the arm, shoulder and hand) [corrected]. The Upper Extremity Collaborative Group (UECG). Am J Ind Med 1996; 29: 602-8. doi: 10.1002/(sici)1097-0274(199606)29:6<602::aid-ajim4>3.0.co;2-l. 8773720

[10] Aasheim T, Finsen V. The DASH and the QuickDASH instruments: normative values in the general population in Norway. J Hand Surg Eur 2014; 39: 140-4. doi: 10.1177/1753193413481302. 23520389

[11] Franchignoni F, Vercelli S, Giordano A, Sartorio F, Bravini E, Ferriero G. Minimal clinically important difference of the disabilities of the arm, shoulder and hand outcome measure (DASH) and its shortened version (QuickDASH). J Orthop Sports Phys Ther 2014; 44: 30-9. doi: 10.2519/jospt.2014.4893. 24175606

[12] Sorensen A A, Howard D, Tan W H, Ketchersid J, Calfee R P. Minimal clinically important differences of 3 patient-rated outcomes instruments. J Hand Surg Am 2013; 38: 641-9. doi: 10.1016/j.jhsa.2012.12.032. 23481405 PMC3640345

[13] van de Water A T, Shields N, Taylor N F. Outcome measures in the management of proximal humeral fractures: a systematic review of their use and psychometric properties. J Shoulder Elbow Surg 2011; 20: 333-43. doi: 10.1016/j.jse.2010.10.028. 21276929

[14] Gummesson C, Atroshi I, Ekdahl C. The disabilities of the arm, shoulder and hand (DASH) outcome questionnaire: longitudinal construct validity and measuring self-rated health change after surgery. BMC Musculoskelet Disord 2003; 4: 11. doi: 10.1186/1471-2474-4-11. 12809562 PMC165599

[15] Hunsaker F G, Cioffi D A, Amadio P C, Wright J G, Caughlin B. The American Academy of Orthopaedic Surgeons outcomes instruments: normative values from the general population. J Bone Joint Surg Am 2002; 84-A: 208-15. doi: 10.2106/00004623-200202000-00007. 11861726

[16] Brooks R. EuroQol: the current state of play. Health Policy 1996; 37: 53-72. doi: 10.1016/0168-8510(96)00822-6. 10158943

[17] Lloyd A, Pickard A S. The EQ-5D and the EuroQol Group. Value Health 2019; 22: 21-2. doi: 10.1016/j.jval.2018.12.002. 30661629

[18] Health TNIoP. Måleinstrumentet EQ-5D. Available from: www.fhi.no/ku/brukererfaringer/sporreskjemabank/maleinstrumentet-eq-5d/, 2020.

[19] NICE. National Institute for Health and Care Excellence, Guide to the Methods of Technology. London: NICE; 2013. 27905712

[20] Stavem K, Augestad L A, Kristiansen I S, Rand K. General population norms for the EQ-5D-3 L in Norway: comparison of postal and web surveys. Health Qual Life Outcomes 2018; 16: 204. doi: 10.1186/s12955-018-1029-1. 30340499 PMC6194590

[21] Olerud P, Tidermark J, Ponzer S, Ahrengart L, Bergström G. Responsiveness of the EQ-5D in patients with proximal humeral fractures. J Shoulder Elbow Surg 2011; 20: 1200-6. doi: 10.1016/j.jse.2011.06.010. 22014617

[22] Ramsey S D, Willke R J, Glick H, Reed S D, Augustovski F, Jonsson B, et al. Cost-effectiveness analysis alongside clinical trials II-An ISPOR Good Research Practices Task Force report. Value Health 2015; 18: 161-72. doi: 10.1016/j.jval.2015.02.001. 25773551

[23] Wittrup-Jensen K U, Lauridsen J, Gudex C, Pedersen K M. Generation of a Danish TTO value set for EQ-5D health states. Scand J Public Health 2009; 37: 459-66. doi: 10.1177/1403494809105287. 19411320

[24] Drummond M F, Sculpher M J, Claxton K. Methods for the economic evaluation of health care programmes. 4th ed. Oxford: Oxford University Press; 2015.

[25] Ministry of Health and Care Services Norway. Principles for priority setting in health care:- summary of a white paper on priority setting in the Norwegian health care sector. In: Services MoHaC, ed. Available from: https://www.Regjeringen.no, 2015-2016.

[26] Plath J E, Kerschbaum C, Seebauer T, Holz R, Henderson D J H, Forch S, et al. Locking nail versus locking plate for proximal humeral fracture fixation in an elderly population: a prospective randomised controlled trial. BMC Musculoskelet Disord 2019; 20: 20. doi: 10.1186/s12891-019-2399-1. 30630465 PMC6329164

[27] Gracitelli M E C, Malavolta E A, Assuncao J H, Ferreira Neto A A, Silva J S, Hernandez A J. Locking intramedullary nails versus locking plates for the treatment of proximal humerus fractures. Expert Rev Med Devices 2017; 14: 733-9. doi: 10.1080/17434440.2017.1364624. 28792243

[28] Konrad G, Audige L, Lambert S, Hertel R, Sudkamp N P. Similar outcomes for nail versus plate fixation of three-part proximal humeral fractures. Clin Orthop Relat Res 2012; 470: 602-9. doi: 10.1007/s11999-011-2056-y. 21879402 PMC3254759

[29] Boyer P, Couffignal C, Bahman M, Mylle G, Rousseau M A, Dukan R. Displaced three and four part proximal humeral fractures: prospective controlled randomized open-label two-arm study comparing intramedullary nailing and locking plate. Int Orthop 2021; 45: 2917-26. doi: 10.1007/s00264-021-05217-9. 34554308

[30] Gardner M J, Weil Y, Barker J U, Kelly B T, Helfet D L, Lorich D G. The importance of medial support in locked plating of proximal humerus fractures. J Orthop Trauma 2007; 21: 185-91. doi: 10.1097/BOT.0b013e3180333094. 17473755

[31] Oppeboen S, Wikeroy A K B, Fuglesang H F S, Dolatowski F C, Randsborg P H. Calcar screws and adequate reduction reduced the risk of fixation failure in proximal humeral fractures treated with a locking plate: 190 patients followed for a mean of 3 years. J Orthop Surg Res 2018; 13: 197. doi: 10.1186/s13018-018-0906-y. 30092807 PMC6085712

[32] Bai L, Fu Z, An S, Zhang P, Zhang D, Jiang B. Effect of calcar screw use in surgical neck fractures of the proximal humerus with unstable medial support: a biomechanical study. J Orthop Trauma 2014; 28: 452-457. doi: 10.1097/bot.0000000000000057. 24662994

[33] Launonen A P, Lepola V, Flinkkila T, Strandberg N, Ojanpera J, Rissanen P, et al. Conservative treatment, plate fixation, or prosthesis for proximal humeral fracture: a prospective randomized study. BMC Musculoskelet Disord 2012; 13: 167. doi: 10.1186/1471-2474-13-167. 22954329 PMC3520878

[34] Launonen A P, Sumrein B O, Reito A, Lepola V, Paloneva J, Berg H E, et al. Surgery with locking plate or hemiarthroplasty versus nonoperative treatment of 3-4-part proximal humerus fractures in older patients (NITEP): an open-label randomized trial. PLoS Med 2023; 20: e1004308. doi: 10.1371/journal.pmed.1004308. 38015877 PMC10683994

[35] Fjalestad T, Hole M O, Hovden I A, Blucher J, Stromsoe K. Surgical treatment with an angular stable plate for complex displaced proximal humeral fractures in elderly patients: a randomized controlled trial. J Orthop Trauma 2012; 26: 98-106. doi: 10.1097/BOT.0b013e31821c2e15. 21804410

[36] Corbacho B, Duarte A, Keding A, Handoll H, Chuang L H, Torgerson D, et al. Cost effectiveness of surgical versus non-surgical treatment of adults with displaced fractures of the proximal humerus: economic evaluation alongside the PROFHER trial. Bone Joint J 2016; 98-b: 152-9. doi: 10.1302/0301-620x.98b2.36614 26850418

[37] Fjalestad T, Hole M, Jørgensen J J, Strømsøe K, Kristiansen I S. Health and cost consequences of surgical versus conservative treatment for a comminuted proximal humeral fracture in elderly patients. Injury 2010; 41: 599-605. doi: 10.1016/j.injury.2009.10.056. 19945102

[38] Hammer O L, Jakobsen R B, Clementsen S, Fuglesang H, Bjornelv G W, Randsborg P H. Cost-effectiveness of volar locking plate compared with augmented external fixation for displaced intra-articular wrist fractures. J Bone Joint Surg Am 2020; 102: 2049-59. doi: 10.2106/jbjs.19.01288. 32947595

